# Investigation of Extreme Response Style in Family Caregiving

**DOI:** 10.1093/geroni/igaf122.3089

**Published:** 2025-12-31

**Authors:** Mustafa Yildiz, Qiyun Liu, Carolyn Pickering

**Affiliations:** Cizik School of Nursing at UTHealth Houston Texas, Houston, Texas, United States; UTHealth Houston, Houston, Texas, United States; UTHealth Houston, Houston, Texas, United States

## Abstract

Extreme Response Style (ERS) is a response bias introducing systematic measurement error because individuals differ in their tendency to select extreme end points when responding to surveys with rating categories. Since caregiving studies heavily rely on self-report measures, ERS may pose significant threats to measurement validity in family caregiving. Understanding its predictors is essential for improving validity of inferences in caregiver populations. While prior research links ERS to intelligence, culture, age, and ethnicity among many other variables, determinants of ERS in family caregiving is an unknown phenomenon. This study extends the prior research by examining ERS predictors in family caregiving using a national sample of caregivers (N = 453). ERS was measured with a latent variable through item response tree models by employing a 10-item scale with 5 response categories ranging from Strongly Disagree to Strongly Agree. Predictors of ERS are demographics, caregiving specific variables, and mental health variables. Findings indicated that caregiver ethnicity, gender, education, age, poverty, family issues, anxiety, depression, avoidant/social coping and hours cared do not predict ERS scores. However, increased emotion dysregulation predicts lower scores in ERS. In contrast, increases in positive health status, number of medications used, and problem-based coping predict higher ERS scores. Lastly, being White determines lower ERS levels compared to non-White participants. These results underscore the importance of accounting for ERS in family caregiving research to improve the validity of self-report measures. Understanding ERS predictors can inform survey designs and statistical corrections, ensuring more accurate assessments of caregiver samples.

